# A novel low light object detection method based on the YOLOv5 fusion feature enhancement

**DOI:** 10.1038/s41598-024-54428-8

**Published:** 2024-02-23

**Authors:** Daxin Peng, Wei Ding, Tong Zhen

**Affiliations:** 1 College of Information Science and Engineering, Henan University of Technology, Zhengzhou, 450001 China; 2 Key Laboratory of Grain Information Processing and Control, Ministry of Education, Henan University of Technology, Zhengzhou, 450001 China; 3 Anhui Jianzhu University, Hefei, 230601 Anhui China

**Keywords:** Low-light scene, Object detection, Image enhancement, Deep learning, Optical sensors, Electrical and electronic engineering

## Abstract

Low-light object detection is an important research area in computer vision, but it is also a difficult issue. This research offers a low-light target detection network, NLE-YOLO, based on YOLOV5, to address the issues of insufficient illumination and noise interference experienced by target detection tasks in low-light environments. The network initially preprocesses the input image with an improvement technique before suppressing high-frequency noise and enhancing essential information with C2fLEFEM, a unique feature extraction module. We also created a multi-scale feature extraction module, AMC2fLEFEM, and an attention mechanism receptive field module, AMRFB, which are utilized to extract features of multiple scales and enhance the receptive field. The C2fLEFEM module, in particular, merges the LEF and FEM modules on top of the C2f module. The LEF module employs a low-frequency filter to remove high-frequency noise; the FEM module employs dual inputs to fuse low-frequency enhanced and original features; and the C2f module employs a gradient retention method to minimize information loss. The AMC2fLEFEM module combines the SimAM attention mechanism and uses the pixel relationship to obtain features of different receptive fields, adapt to brightness changes, capture the difference between the target and the background, improve the network's feature extraction capability, and effectively reduce the impact of noise. The AMRFB module employs atrous convolution to enlarge the receptive field, maintain global information, and adjust to targets of various scales. Finally, for low-light settings, we replaced the original YOLOv5 detection head with a decoupled head. The Exdark dataset experiments show that our method outperforms previous methods in terms of detection accuracy and performance.

## Introduction

In recent years, artificial intelligence technology has advanced significantly, and object detection as a fundamental problem in computer vision has become widely used. For example, in the realm of autonomous driving^1^, it is employed for 3D object detection^2^. Target detection is accomplished in real time and with excellent precision. In the realm of video surveillance^3^, it combines adaptive background modeling to detect moving objects in videos. Furthermore, target detection technology is widely used in medicine, remote sensing, and other domains.

Target detection techniques emphasize target items to be discovered using image processing techniques, and the majority of current target detection techniques use high-quality images obtained from image collection equipment as data sets for research. However, high-quality, crisp photographs are frequently difficult to get in practical applications. Due to the limitations of the equipment in low-light environments, the captured images will be underexposed, with color distortion, low contrast, and signal-to-noise ratio degradation, which seriously affects the accuracy of target detection and is also a major challenge for the target detection task.

Some researchers try to enhance hardware devices to overcome the problem of low target detection accuracy in low-light circumstances; however, this strategy is expensive and difficult to implement broadly. As a result, additional researchers began to strive to improve target identification algorithms to make them more adaptable to low-light conditions. Some researchers have enhanced detection performance by restructuring the target detection network so that it can extract more critical information. Literature^4^, based on RFB-Net^5^, employs a feature pyramid network and a context fusion network to perform target identification under low-light situations, which improves the network's feature extraction capability. Although the aforementioned method solves the problem to some extent, in excessively complicated situations, such as extremely low-light conditions, it is difficult to identify the foreground from the background of an image, resulting in a degradation of the detection effect. The researchers employed a low-light picture enhancement technique for preprocessing to overcome the aforementioned concerns. In the literature^6^, color adjustment is performed on low-light photos for future detection. Literature^7^ coupled the physical lighting model with a deep neural network to improve image quality and get good results in the target detection test. The literature^8^ developed an image enhancement network that is better suited for low-light conditions, and when paired with the target detection network, it effectively enhanced detection accuracy. Although the enhancement method significantly improves brightness, it also amplifies noise and causes artifacts during the enhancement process, which is obviously not suitable for target detection.

To solve the aforementioned difficulties, we propose the NLE-YOLO low-light object detection model. We use the improved image as input in our model to extract more information for better results. Given that the C2f module allows the model to get richer gradient flow information, we propose a novel feature extraction module based on C2f, C2fLEFEM, that can suppress high-frequency noise and enhance essential information. To reduce the influence of noise and acquire richer feature information, we utilize this module to replace the C3 module in the original YOLOv5 network, except for the last three in the neck. Furthermore, we propose a new Attentional Receptive Field Block (AMRFB) inspired by RFB^5^. This module can broaden the receptive field and allocate different attention domains to distinct receptive fields, boosting the network's feature extraction ability. Because the input may have extreme values in low-light settings, we replaced the SPPF module with the SimSPPF module to achieve faster inference speed and stronger feature representation capabilities. Following that, we replaced the three C3 modules in the neck with the AMC2fLEFEM module, which merged the Simple Attention Module (SimAM)^9^ on the basis of the feature extraction module C2fLEFEM to extract features of various scales, causing the network to pay greater attention to them. The target area decreases the influence of noise and increases the network's feature-learning capabilities. Finally, we replaced the initial detecting head with a decoupled head, which improves the network's performance on the ExDark dataset and makes it more appropriate for low-light conditions.

Our contributions are summarized as follows:

1. We propose a novel low-light object identification network, NLE-YOLO, and a novel feature extraction module, C2fLEFEM, to suppress high-frequency noise and enhance essential information. We combined the simple attention module (SimAM) with the feature extraction module C2fLEFEM to extract features of different scales, allowing the network to pay more attention to the target area, reduce the influence of noise, and improve the network's feature learning ability.
2. We propose a new Attentional Receptive Field Block (AMRFB). This module may enlarge the receptive field and assign different attention domains to distinct receptive fields, greatly boosting the network's feature extraction ability.
3. In order to adapt to the low-light object detection task, we substitute the original detection head with a decoupled head. At the same time, we replaced the SPPF module with the SimSPPF module, which increases the network's inference performance and feature extraction ability.
4. Compared with other state-of-the-art methods, NLE-YOLO shows good performance on the low-light dataset ExDark.

## Related work

### Object detection

There are three types of existing object detection algorithms: one-stage algorithms, two-stage algorithms, and anchor-free methods. YOLO and SSD are examples of one-stage algorithms. RCNN, Fast RCNN, and Faster RCNN are two-stage algorithms.

One-stage detection algorithms: One-stage detection algorithms use only one feature extraction to complete the detection, which is faster but less accurate. The primary representatives are YOLO (You Only Look Once)^10^, SSD^11^, and RetinaNet^12^. YOLOv1 was proposed in the literature^13^ in 2016. The program separates the image into numerous grids and forecasts each grid. This approach is fast but has poor detection accuracy. YOLOv2^14^ has significantly improved in accuracy and speed when compared to YOLOv1. YOLOv2 employs DarkNet19 as a feature extraction network and combines training with target classification and detection to boost accuracy. However, YOLOv2 has just one detection branch, making it difficult to gather multi-scale context information. YOLOv3^15^ differs from YOLOv2 in that it employs DarkNet53 as a feature extraction network and FPN to handle multi-scale detection difficulties. However, there are issues with incorrect positioning. YOLOv4^16^ was proposed as a result of this. It has made significant changes and performs well when compared to the previous edition. Then there were YOLOv5, YOLOv7^17^, and YOLOv8. YOLOv5 is now in a relatively stable state. As a one-stage detection method, the SSD algorithm features several detection branches that may recognize targets of varying sizes and efficiently enhance accuracy. The RetinaNet algorithm is a high-precision target detection technique that employs focal loss to address the issue of imbalance between positive and negative samples.

Two-stage target detection algorithm: The two-stage target detection algorithm necessitates two feature extractions and predictions. RCNN^18^ is the first effort to apply deep learning to object detection. A selective search technique is utilized to choose potential boxes first, and then features are retrieved and predicted. When compared to the previous method, its accuracy is greatly enhanced, but there will be feature redundancy, which will slow down the network's performance. Fast RCNN^19^ is an upgraded form of RCNN. It initially pulls features from the entire image, then uses ROI pooling and passes it to the fully connected layer to extract higher-level features, and lastly uses the Softmax layer to predict. This method eliminates the problem of recurrent feature extraction and significantly improves detection speed, but it still relies on the selective search algorithm to generate candidate regions, which has an impact on accuracy. Faster RCNN^20^ improves on Fast RCNN. This approach replaces the selective search algorithm with the RPN network to generate candidate regions, significantly improving detection speed and accuracy. Cascade RCNN^21^ enhances Faster RCNN by cascading the detection elements to improve detection accuracy.

Anchor-free algorithm: This method does not employ anchor boxes and instead detects key spots. CornerNet^22^ is the anchor-free algorithm's ground-breaking work. This method simplifies object detection by converting it to key point detection without the use of anchor boxes. However, this technique only concentrates on the most important points and ignores the target's internal information. CenterNet^23^ focused on detecting the target's center point and developed cascaded corner pooling and center pooling modules to boost accuracy. However, this approach is poor at distinguishing overlapping items. Based on this, the FASF^24^ network, which employs the FASF module to realize autonomous learning selection characteristics, is presented.

### Low light image enhancement

Low-light image enhancement seeks to recover detailed photos from low-light circumstances and deliver high-quality images for computer vision tasks such as object detection. Guo et al.^25^ introduced a new reference-free depth curve estimation (Zero DCE) method that uses a lightweight deep network, DCE-Net, to adapt the dynamic range of a given image and does not require the preparation of paired data in advance. However, this method ignores the effect of noise, and the network model is vast and time-consuming. Li et al.^26^ also released Zero DCE +  +, an accelerated and lightweight version of Zero DCE that has rapid processing speed while keeping Zero DCE's enhanced performance. Ni et al.^27^ introduced a new recurrent interactive generative confrontation network (CIGAN) for unsupervised low-light picture augmentation that is divided into three components and incorporates an attention strategy to increase image quality. Wang et al.^28^ proposed MAGAN, a hybrid attention mechanism-guided generative adversarial network that uses a hybrid attention layer to understand the link between pixels and images in order to improve images while reducing noise. Qiao et al.^29^ proposed a generative adversarial network method based on the anti-attention mechanism, which removes noise using the deep aggregation pyramid pooling module combined with multi-scale background information and eliminates chromatic aberration using the anti-attention module to improve the quality of enhanced images. A semantically guided low-light image improvement network was proposed in the literature^30^ (SGZ). The approach calculates illumination using depth-wise separable convolutions and uses a semantic segmentation network to preserve semantic information.

### Target detection under complex conditions

Detecting objects in complex environments is critical for some activities, such as autonomous driving, video monitoring, and so on. Many strategies for adapting to target detection in difficult conditions have been presented. The literature^31^ constructed a novel color scale offset compensation model to perform adaptive color scale correction on the image in order to increase the detecting capacity of autonomous driving equipment in severe situations. This approach improves target detection accuracy while also enhancing target clarity in tough situations. Literature^32^ designed a multi-level functional cross-integration module and attention refinement module to overcome the problem of poor detection effects in low-light settings. The literature^33^ considers low-light target identification to be a domain adaptation problem and presents a generative model that converts normal-light domain images to low-light domain images and adapts the network to low-light settings. Similarly, literature^34^ presents a data domain transfer-based training strategy that fuses a large-scale normal illumination dataset with a small number of low-light datasets and combines low-light enhancement and attention processes to improve low-light circumstances. In terms of object detection precision. The literature^35^ proposed a foggy, weather-adapted target identification framework, IDOD-YOLOv7. This method combines the image defogging module (AOD), the image enhancement module (SAIP), and the YOLOv7 detection module to significantly increase target detection in hazy circumstances. Furthermore, the literature^36^ suggests IA-YOLO, a new adaptive image object identification approach for adapting to object detection under challenging conditions. This method, in particular, proposes a differentiable image processing module (DIP) and a tiny convolutional neural network for parameter prediction (CNN-PP). CNN-PP can effectively modify the DIP settings to enhance the image, allowing it to handle photos under tough conditions adaptively.

## Method

### Network structure

The YOLOv5 target detection technique is a one-stage detection system based on regression. It is faster than other detection methods, has less background interference, and has a broader range of applications. It is now in a relatively stable state. Given the detection accuracy and the experimental equipment, the YOLOv5l model is chosen as the baseline model in this research.

**Figure 1:**
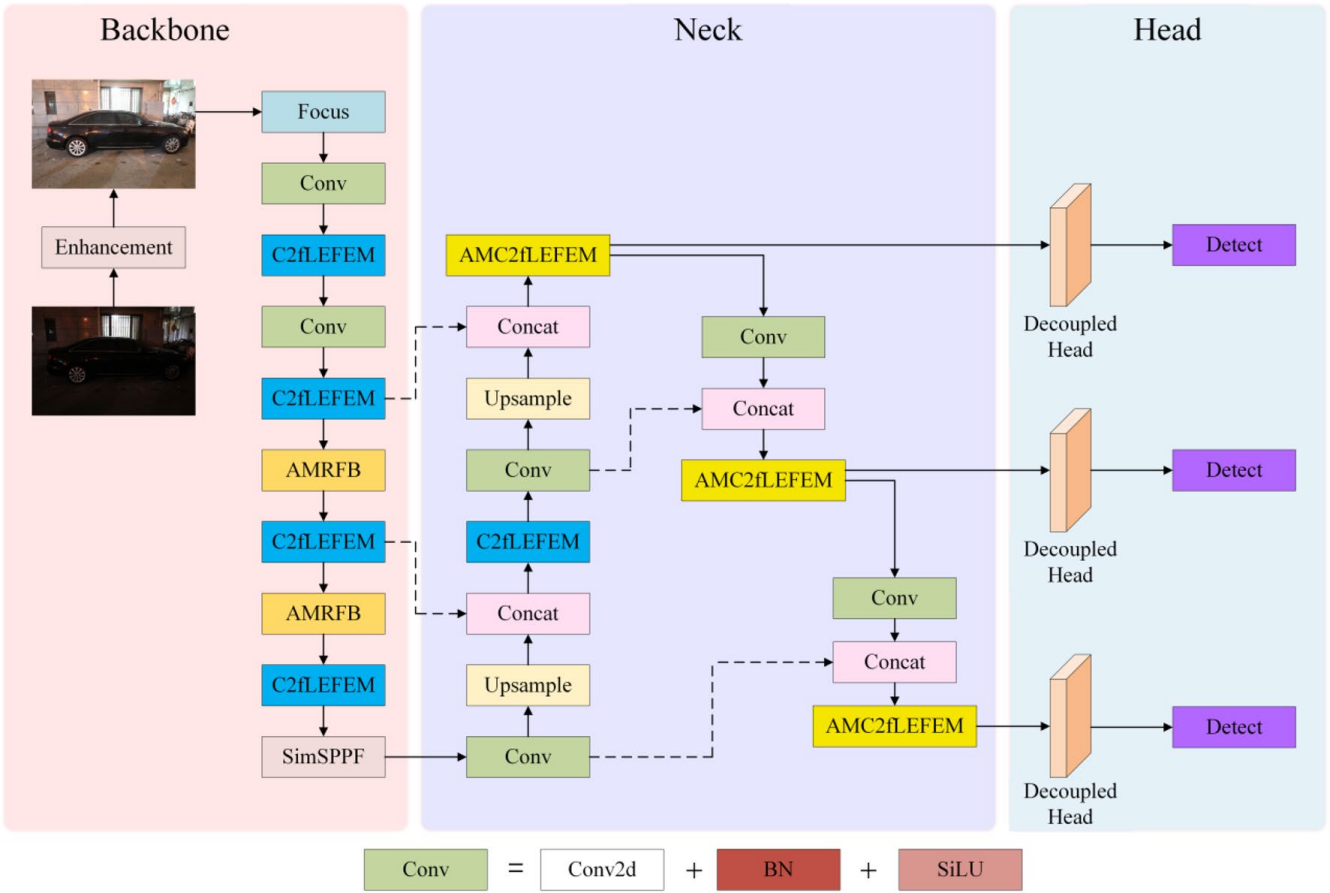
Network structure diagram.

Images acquired in low-light situations have poor visibility due to low-light interference, low contrast, and noise, which reduces detection effectiveness. In order to address the aforementioned issues and increase detection performance while drawing inspiration from literature^37,38^ and^39^, we propose the NLE-YOLO network, a revolutionary low-light object identification network. Figure 1 shows the structural diagram.

As illustrated in Fig. 1, we used our newly created C2fLEFEM module to replace the C3 module in the backbone as well as the initial C3 module in the neck. Our C2fLEFEM module can suppress high-frequency noise while increasing features, thereby improving the network's feature extraction performance. We integrated the simple attention module (SimAM) with the feature extraction module C2fLEFEM to extract features of different scales so that the network may pay more attention to the target area and limit the influence of noise. We created an AMRFB module to replace the last two convolutional layers in the backbone component of our network layout, maximizing feature information retention. We replaced the original detection head with a decoupling head and separated the classification task from the detection task because the original detection head shared the parameters of the two tasks of object localization and object classification, which would affect detection accuracy. Furthermore, we replaced the SPPF module with the SimSPPF module to achieve quicker inference speed and better feature representation ability.

### C2fLEFEM module

**Figure 2:**
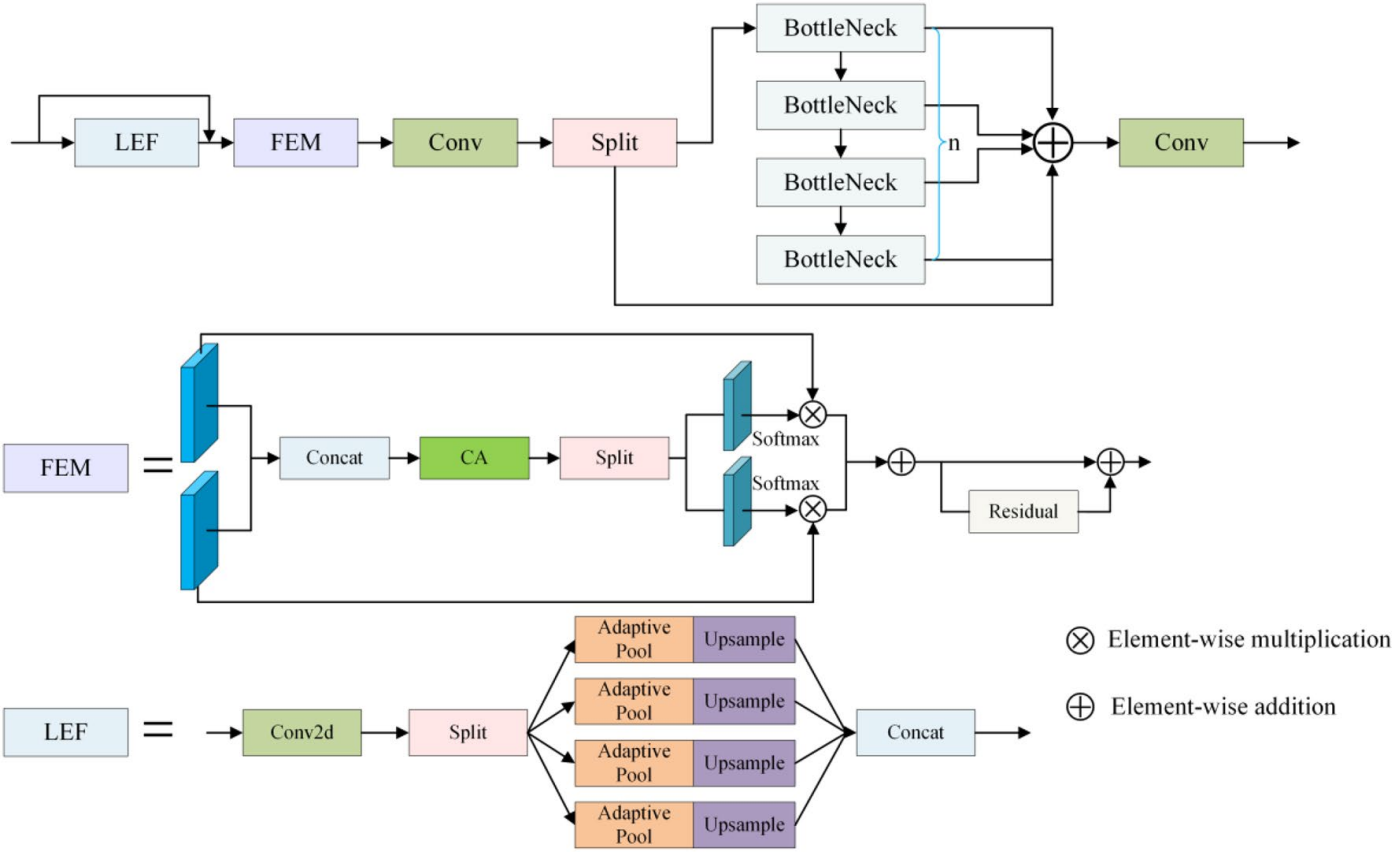
Diagram of the C2fLEFEM module structure.

In the original YOLOv5 network, the C3 module is employed to reduce model complexity while increasing the receptive field. However, in low-light situations, the image's brightness and contrast are insufficient, and even noise is mixed, and the C3 module is unable to extract useful feature information and limit the influence of noise, which obviously impairs detection performance. In order to solve this problem, we designed a network inspired by C2f and SKNet^40^, considering that low-frequency components often contain a large amount of semantic information in images and noise information often corresponds to high-frequency information. The C2fLEFEM module is responsible for enhancing feature semantic information. Figure 2 depicts a specific structure diagram.

The C2fLEFEM module, as shown in Fig. 2, is composed of three sub-modules: the Low Frequency Filter Enhancement Module (LEF), the Feature Enhancement Module (FEM), and the C2f module. To begin, the LEF module applies low-frequency filtering to the input features in order to remove high-frequency noise. The FEM module then accepts the low-frequency enhanced features and the original features as dual inputs and keeps the original low-frequency semantic information while reducing high-frequency noise through a series of procedures. Finally, the FEM module's output is processed further by the C2f module to preserve more gradient information and reduce information loss.

The low-frequency components have the majority of the semantic information in the image and are the main information in the detection task among the components at each scale. We employ the LEF module to gather more low-frequency information in order to improve the semantic content of the image. We filter the acquired low-frequency information using adaptive mean pooling techniques of varying sizes. The FEM module then learns the weights of each feature channel, allowing the network to adaptively fuse the original low-light information with the low-frequency semantic features to obtain richer semantic features. Finally, the feature fusion is accomplished using the C2f module, and the gradient information is maintained.

SKNet is a selective kernel-based network structure that dynamically modifies the size of each channel's receptive field to adapt to varied workloads and conditions. The core idea of SKNet is to use the attention mechanism to select the best kernel function to achieve adaptive feature fusion. Inspired by SKNet, we created the submodule FEM to improve semantic characteristics. We compare the submodule FEM with SKNet to assess the similarities and differences between the C2fLEFEM module and SKNet. The similarities between our module and SKNet are that both sub-module FEM and SKNet use the concept of segmentation and post-processing to quickly learn the weights of each feature channel to capture relevant information. Unlike SKNet, which employs the concepts of segmentation, processing, and selection, the sub-module FEM employs the concepts of segmentation, processing, and noise reduction. The steps of processing differ among them. Specifically, following segmentation, SKNet sums the input features to achieve feature fusion. Given the effect of noise in this assignment, merely summing the input features will result in the end features being affected by noise, so we use feature splicing to achieve feature fusion. In the final stage, unlike SKNet's selection of distinct spatial scale information, the sub-module FEM suppresses the noisy channels so that the network pays more attention to the channel where the goal information is located, and residual connections are established to prevent network degradation.

Assuming input feature $I \in R^{{H \times W \times C_{1} }}$, the LEF module is the first to process it. The size of the input feature is initially reduced to $I \in R^{{H \times W \times C_{2} }}$ in the LEF module, where $C_{1}$ is the original channel number and $C_{2}$ is adaptively altered based on the channel number of each layer. Following that, we employ adaptive pooling to suppress high-frequency noise while capturing low-frequency semantic information. We use $1 \times 1$, $2 \times 2$,$3 \times 3$, and $6 \times 6$ adaptive average pooling modules to account for the various frequency thresholds of distinct semantic information. At each scale, an upsampling module is employed to restore the original feature information. Finally, the collected features are combined to achieve information fusion. Formula (1) depicts the exact implementation procedure.

1 $$\mathop {H(I_{i} )}\limits_{{i \in \{ 1,2,3,4\} }} = Concat(Up(\alpha_{k} (I_{i} )))$$

where $I_{i}$ represents the $i$-th input, $Concat$ represents feature splicing,$Up$ represents upsampling, and $a_{k}$ represents the adaptive mean pooling operation of size $k \times k$.

It is passed to the FEM feature enhancement module after being processed by the LEF module. We begin by fusing the improved low-frequency features with the original input features in this module. The channel's weight vector is then calculated by the CA module, and the vector is separated into two halves, with the two inputs weighted and calibrated separately. Finally, the feature vectors are inserted pixel by pixel, and residual connections are provided to prevent gradient dispersion. Assuming that this module's input feature is $M \in R^{{H \times W \times C_{2} }}$, the feature after feature fusion is $F \in R^{{H \times W \times 2C_{2} }}$. The channel vector acquired after the CA module's processing is $L \in R^{{1 \times 1 \times 2C_{2} }}$. Formulas (2–3) can be used to compute the channel vector.

2 $$L = G(F,W)$$

3 $$G(F,W) = \sigma (W_{2} (\delta (W_{1} (AAP(F))))$$

where,$W$, $W_{1}$, and $W_{2}$ are learnable parameters. The sigmoid function is represented by the number $\sigma$. The ReLU function is represented by the number $\delta$. The adaptive average pooling layer is represented by layer $AAP$. The resultant channel vector is then separated into two sections, and the input features are weighted and calibrated to reduce the influence of noise and direct the network's attention to critical feature information. Finally, the characteristics gathered are forwarded to the C2f module for processing to get the final result.

### AMC2fLEFEM module

**Figure 3:**
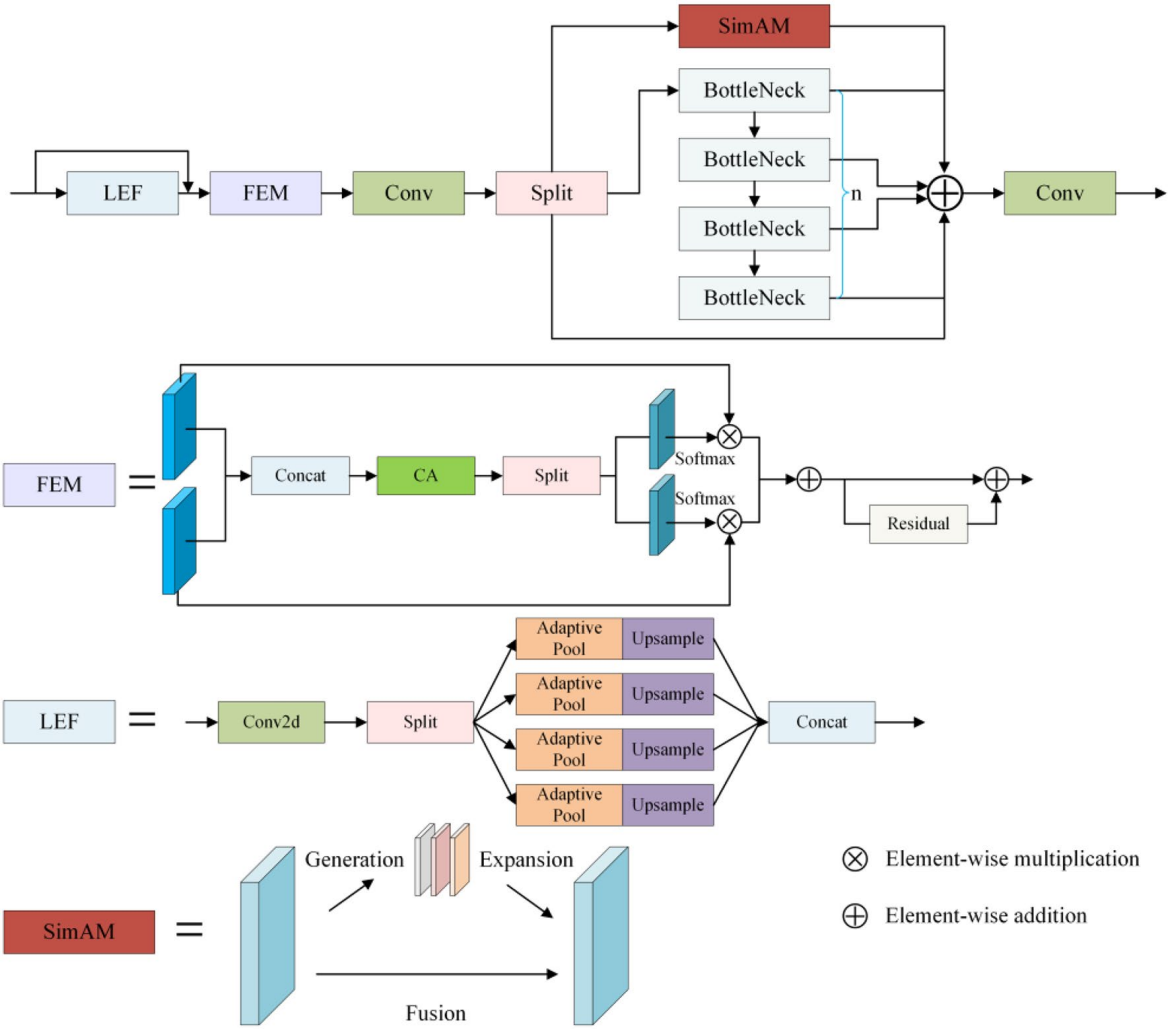
Diagram of the AMC2fLEFEM module structure.

The last three C3 modules in the YOLOv5 network's neck are upsampling and fusing modules, but they merely perform simple addition operations to fuse features, ignoring the relationship and weight values across feature maps. Furthermore, in low-light conditions, the image may be contaminated with noise and the brightness may be poor, affecting the detection effect. To address these issues, we present the SimAM attention mechanism, which is based on the modules proposed in Section "C2fLEFEM module", as well as a novel feature fusion extraction module, AMC2fLEFEM. The network's feature extraction capability is enhanced, and the influence of noise is successfully decreased. Figure 3 depicts its structural diagram.

The AMC2fLEFEM module combines the learned features with the C2f convolution branch to gain more gradient features and semantic information. This module combines the SimAM attention mechanism with the extraction of low-level features. It is worth noting that this is not merely using an attention mechanism. Our innovation is that we created the LEF and FEM modules to extract and enhance semantic features, as well as a feature learning branch with an attention mechanism, so that the network can efficiently extract low-level features while decreasing the impact of noise. Finally, feature fusion is conducted on this branch with other branches to obtain richer semantic characteristics.

The SimAM attention module is introduced in this module. The attention module is a non-parameter, three-dimensional weight attention module that may focus on crucial information without the use of extra parameters. SimAM attention uses the relationship between different pixels to obtain key features under different receptive fields, particularly in low-light conditions where the image's brightness and contrast are insufficient. The above mechanism can well adapt to brightness changes and effectively capture the target. The distinction between the background and the background in order to extract crucial information more effectively. This attention mechanism calculates weights expressing the relevance of pixels using an energy function, as indicated in Eq. (4).

4 $$e_{t} \left( {w_{t} ,b_{t} ,{\mathbf{y}},x_{i} } \right) = \left( {y_{t} - \hat{t}} \right)^{2} + \frac{1}{M - 1}\sum\limits_{i = 1}^{M - 1} {\left( {y_{o} - \hat{x}_{i} } \right)^{2} }$$

where, $\hat{t} = w_{t} + b_{t}$ and $\hat{x}_{i} = w_{t} x_{i} + b_{t}$ are linear transformations of $t$ and $x_{i}$, $t$ and $x_{i}$ are the target and input features, respectively.$w_{t}$ and $b_{t}$ represent weights and biases, respectively. $M = H \times W$ represents the total number of neurons.

### AMRFB module

**Figure 4:**
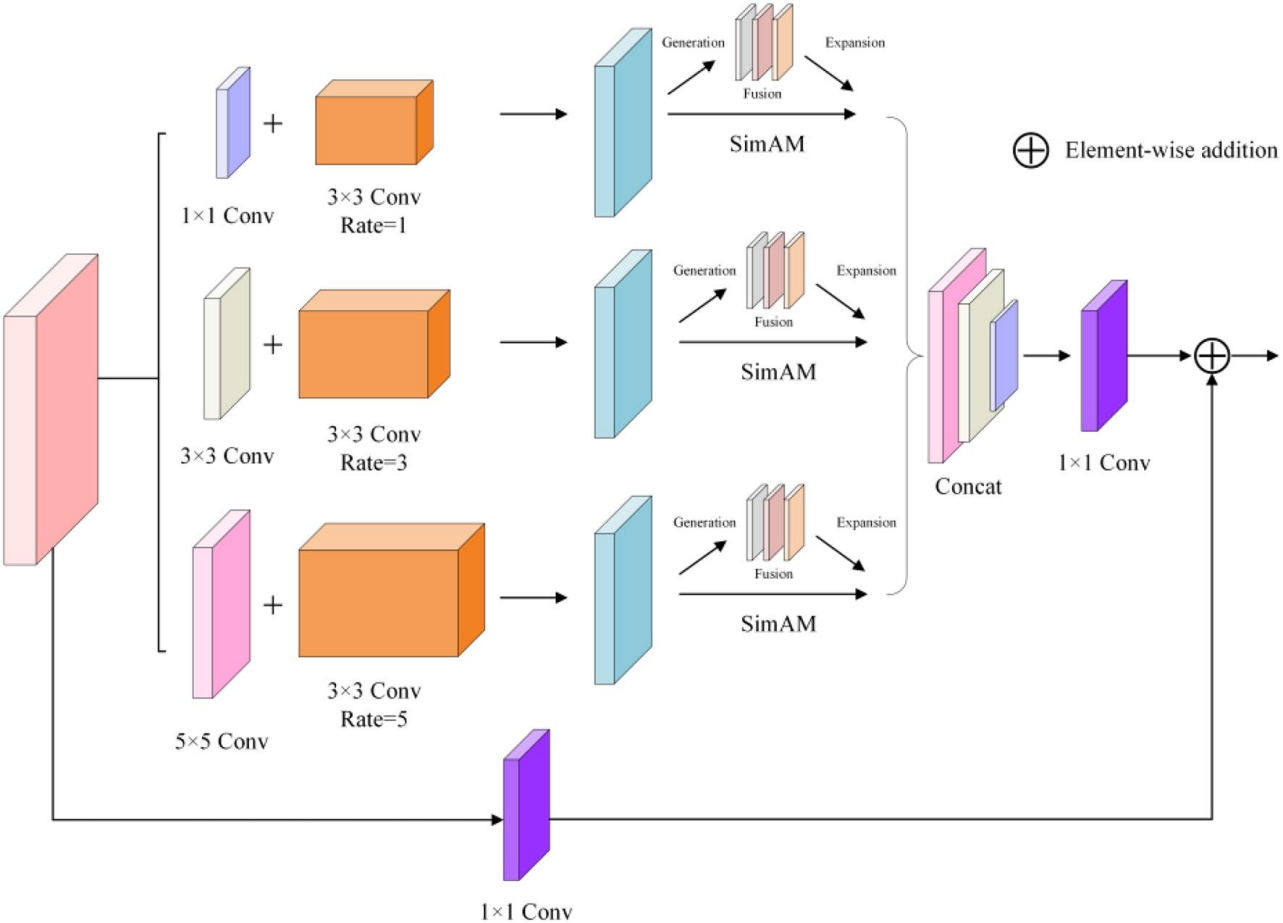
AMRFB module structure diagram.

Ordinary convolutional layers struggle to extract useful features in the original YOLOv5 network because of low-light image contrast and substantial noise and blur issues. How to simultaneously increase the receptive field and focus on crucial target areas is an important issue in low-light target identification jobs. The receptive field is the area size of the input image corresponding to each output unit in the convolutional neural network, which influences the range of information that the network can acquire. Extending the receptive field can help increase perceptions of long-distance information and global semantic understanding. However, increasing the sensory field might generate issues such as information sparsification, loss of details, and additional processing, which can cause the network to fail to focus on crucial target areas. As a result, overcoming the interference of low-light circumstances and maintaining focus on essential target areas while increasing the receptive field is a difficulty that must be overcome. To address this issue, we offer a unique module, AMRFB, for feature information extraction. We integrate the unique attention mechanism SimAM on the basis of RFB, which enhances the receptive field while directing the network's attention to critical target locations, effectively increasing the network's feature extraction capability. Figure 4 depicts the structure diagram of this module.

Given that extending the receptive field will blur the target, which is undesirable for some smaller targets, we replaced the convolution of the medium target and large target detection layers with the AMRFB module in the backbone network.

As shown in Fig. 4, the AMRFB module we built employs atrous convolution, which efficiently maintains more global information while extending the receptive field. Using varied dilation rates, adapt the network to objects of varying scales. We introduce SimAM, a novel attention mechanism, to make the network pay greater attention to effective target regions. In comparison to attention mechanisms such as CBAM, SE, ECA, and others, this attention mechanism does not require any additional parameters to calculate weights. By adding the SimAM attention mechanism, the network can efficiently capture the difference between the target and the background in the low-light image and pay more attention to the critical information, resulting in a stronger feature extraction capacity.

The following factors explain why the AMRFB module can broaden the receptive field while focusing on critical target areas: First, different scale features are produced using different expansion rates, and distinct scale information is collected and used using different scale features. This increases the size and variety of the receptive field. Second, using the SimAM attention mechanism, the attention to different channels and locations may be dynamically altered, highlighting critical target information and capturing the difference between the target and the background. Finally, cross-scale feature fusion allows for feature complementation and improvement, which improves feature quality and expressive ability.

Inspired by literature^41^, we replace the detection head of the original network with a decoupling head^42–44^. The decoupling head assigns classification and regression tasks to distinct network branches and uses different weight values. This can improve detection performance by better distinguishing the foreground and background in low-light settings.

### SimSPPF module

**Figure 5:**
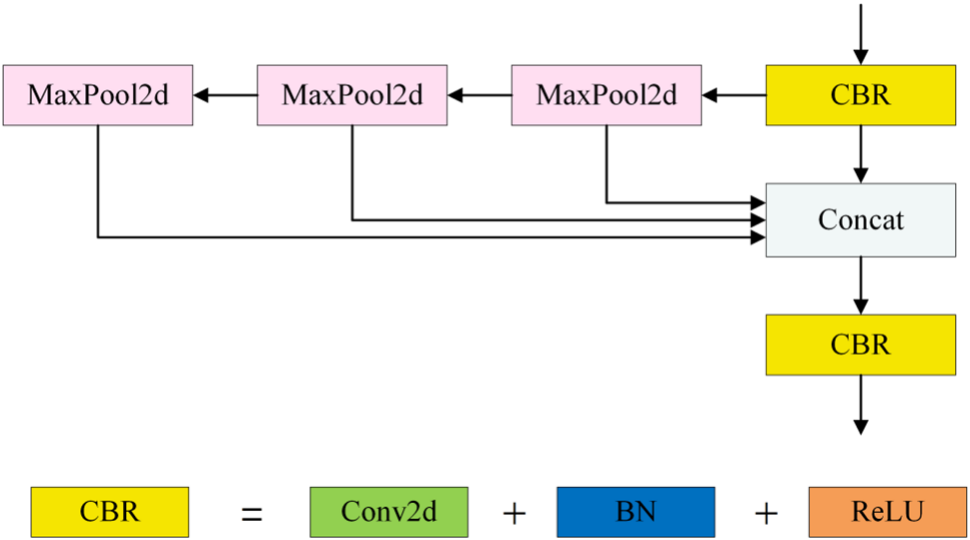
SimSPPF module structure diagram.

To increase model speed, the YOLOv5 network employs the SPPF module and the SiLU function as the activation function. However, if the input image contains extreme values in low-light settings, the SiLU function may produce vanishing or expanding gradient issues. To address this problem, we employ the SimSPPF module in place of the SPPF module. Figure 5 depicts the structural diagram of the SimSPPF module.

## Experimental results

### Experimental details

The experiment makes use of an Intel (R) Xeon (R) Platinum 8350C processor, an RTX 3090 graphics card, and the Linux operating system. Python 3.8, Cuda 11.3, Pytorch, and version 1.10.0 are the software environments. The training parameters are critical to the model's convergence speed and generalization capabilities. As a result, in order to obtain an optimal set of training parameters for the experiments, these parameters must be dynamically updated so that the model achieves the best performance. During the experiments, we employ the control variable approach to alter these parameters, which include the optimization strategy, starting learning rate, momentum, and weight decay. In addition, we examined hardware characteristics such as batch size. We assess convergence speed and model performance by examining metrics graphs and training loss graphs. Based on these evaluations, we arrived at the following parameter settings: In the experiment, the SGD optimizer is employed, with an initial learning rate of 0.01, momentum set to 0.937, and weight decay set to 0.0005. During training, mosaic data augmentation is employed, with the batch size set to 16 and the picture size set to $256 \times 256$. We train for 200 epochs in order to avoid local optima. We analyzed many enhancement methods in Section "Evaluation Metrics" and ultimately chose SGZ as the picture improvement approach for our method.

### Experimental dataset

We chose the low-light object detection dataset Exdark as the main dataset to test our method's performance in low-light situations. The Exdark dataset^45^ is a low-light object detection dataset that contains 7363 low-light photos in 10 different situations. It is organized into 12 different categories, each of which covers a different object or situation. Allows for full algorithm evaluation in low-light circumstances. In the experiment, we divide the data set into three parts: training, verification, and testing, in the ratio of 6:2:2.

### Loss functions

The loss function of YOLOv5 is made up of three parts: bounding box regression loss, classification loss, and target loss. Among them, the loss function is determined as follows:

5 $$L = L_{box} + L_{cls} + L_{obj}$$

where, $L_{obj}$ is determined using the $CIoU$ loss function, and the formula for $CIoU$ is as follows:

6 $$L_{CIoU} = 1 - IoU + \frac{{\rho^{2} (b,b^{gt} )}}{{c^{2} }} + av$$

7 $$a = \frac{v}{1 - IoU + v}$$

8 $$v = \frac{4}{{\pi^{2} }}(\arctan \frac{{w^{gt} }}{{h^{gt} }} - \arctan \frac{w}{h})^{2}$$

where, $\rho^{2} (b,b^{gt} )$ is the Euclidean distance between the genuine bounding box and the anticipated bounding box. $c$ is the diagonal length of the smallest enclosing rectangle of the genuine bounding box and the predicted bounding box. $v$ is the distance between the aspect ratios of the ground truth and forecasted bounding boxes. $a$ is the weight coefficient. $w$ is the width of the estimated bounding box. $h$ is the estimated bounding box height. $w^{gt}$ is the width of the genuine bounding box, and $h^{gt}$ is its height.

### Evaluation metrics

We employ P (Precision), R (Recall), mAP (mean Average Precision), and FPS (Frames Per Second) as evaluation metrics to assess the performance of our model.

The fraction of correct detections among all detection findings is referred to as accuracy. It is defined as the ratio of true positive cases (objects belonging to the target category are correctly detected as belonging to the target category) to the sum of true positive cases and false positive cases (objects not belonging to the target category are incorrectly detected as belonging to the target category). The higher the accuracy value, the more effective the model. Equation (9) illustrates this.

9 $$Precision = \frac{TP}{{TP + FP}}$$

The recall rate is defined for a certain category as the ratio of projected positive samples to all positive samples. It is specifically shown in Eq. (10).

10 $$Recall = \frac{TP}{{TP + FN}}$$

We utilize the mAP to express the average precision across all classes, which is the average of the AP for each class.

The average precision of each category is referred to as AP, and it is used to assess the model's correctness for each category. The region enclosed by the PR curve is defined as the AP value, and the larger the value, the better the performance. It is specifically demonstrated in Eq. (11).

11 $$AP = \int_{0}^{1} {P(R)dR}$$

mAP stands for the average precision across all classes. mAP shows the model's performance across all categories, and the higher the value, the better the performance of a particular kind. It is specifically illustrated in Eq. (12).

12 $$mAP = \sum\limits_{i = 1}^{n} {AP_{i} }$$

where $n$ represents the total number of categories and $AP_{i}$ represents the AP value of the $i$-th category.

The frame rate per second (FPS) specifies how many images the object detection network can handle per second. Equation (13) depicts its calculating formula.

13 $$FPS = \frac{N}{T}$$

where $N$ is the number of photos processed and $T$ represents the overall processing time.

### Comparison with state-of-the-art methods

We undertake comparative experiments on the low-light dataset Exdark to validate the efficiency of our technique.

Quantitative evaluation. First, we compare our method to other augmented models. Table 1 shows the results. Among them, mAP@0.5 reflects the average accuracy of all categories when the IoU (Intersection over Union) criterion is 0.5. mAP@0.5:0.95 represents the average mAP at different IoU (0.5–0.95, step size 0.05) criteria. As seen in Table 1, our technique yields significant performance improvements, demonstrating its superiority. Among these, the SGZ algorithm paired with our technique produces the best results. When compared to the original YOLOv5l model, the P value grew by 1.5%, the R value increased by 0.3%, the mAP@0.5 value increased by 1.7%, and the mAP@0.5:0.95 ratio climbed by 1.5%. The value rose by 2.7%. The SGZ algorithm most likely preserves more semantic information while predicting the pixel-level light insufficiency of low-light photos, whereas our method efficiently reduces noise while boosting features. Although the mAP@0.5 value of the URetinexNet algorithm and our method is 0.5% higher than that of the SGZ algorithm and our method, our method runs faster than URetinexNet + NLE-YOLO. The running time of our method is only half that of URetinexNet + NLE-YOLO, and the number of frames per second (FPS) of our method is more than four times its FPS, which is obviously necessary for time-critical applications. indispensable.

**Table 1:**
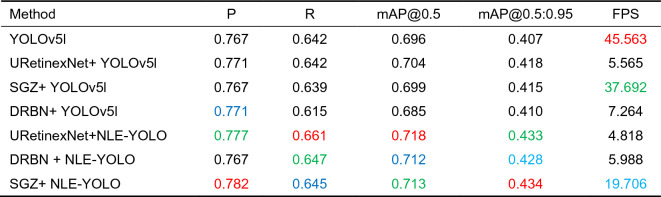
Comparison of our method with several enhancement models (Higher metric values reflect higher performance. Red, green, and blue represent the top-three indicator values, respectively).

**Figure 6:**
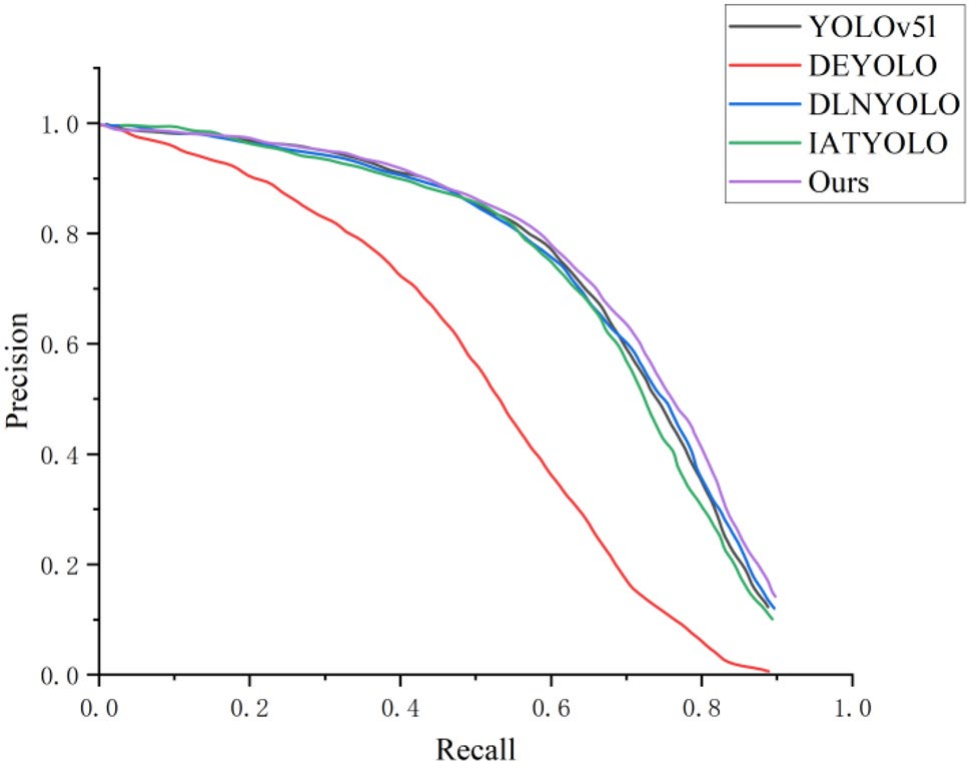
Comparison of PR curves.

**Figure 7:**
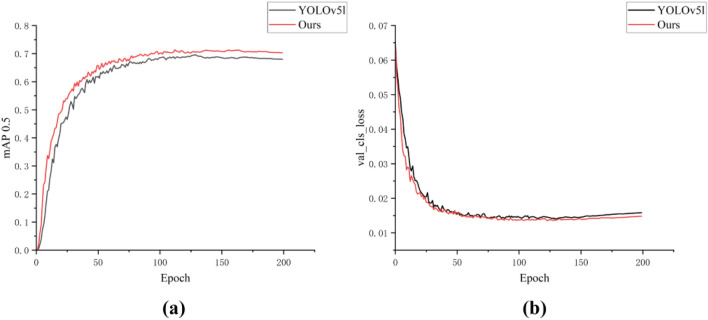
Training mAP and loss comparison. (**a**) Training mAP; (**b**) Training loss.

In addition, we visualized the training results, as shown in Figs. 6 and 7. Figure 6 compares the PR curves of the models engaged in the comparison. It can be seen that the area enclosed by our model's PR curve is the greatest, implying that our method has the best performance.

Figure 7a shows that our technique consistently maintains a higher mAP0.5 value than the original YOLOv5l model. The increase in mAP value indicates that our approach has increased detection accuracy, proving its superiority even more. The training loss in Fig. 7b shows that our technique has lower loss and faster convergence than the original YOLOv5l model. It also demonstrates that our model has superior model learning and optimization capabilities.

To demonstrate our method's superiority, we compare it to other cutting-edge approaches (DE-YOLO^46^, DLN-YOLO^47^, and IAT-YOLO^48^), and the results are provided in Table 2. Table 2 shows that our method has the highest index value, demonstrating its superiority. DLN-YOLO has the best effect among the approaches in the comparison, with a P value of 0.779, a R value of 0.621, a mAP@0.5 of 0.698, and a mAP@0.5:0.95 of 0.412. Our approach has a P value of 0.782, a R value of 0.645, a value of mAP@0.5 of 0.713, and a value of mAP@0.5:0.95 of 0.434, which is 0.3%, 2.4%, and 1.5% greater than DLN-YOLO. When compared to the baseline model, it improves by 1.5%, 0.3%, 1.7%, and 2.7%. The explanation for the increase in index value could be that we incorporated the denoising module and enlarged the receptive field to collect additional semantic information from low-light images.

**Table 2:**
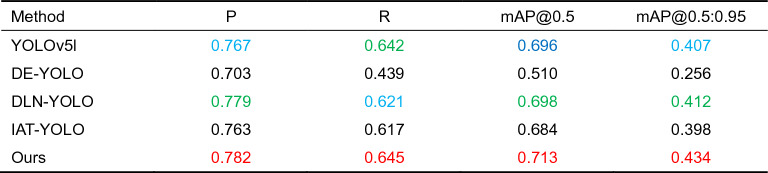
Comparison of our method with state-of-the-art methods (Higher metric values reflect higher performance. Red, green, and blue represent the top-three indicator values, respectively).

In addition to the above evaluation indications, the algorithm's efficiency should be examined, ensuring a full evaluation of the algorithm.

Table 3 compares our method to other methods in terms of FLOPs. Table 3 demonstrates that our method has a larger FLOPs metric value than other methods and needs more computer resources. This is because adding individual modules increases the computational resources of the model. The comparison of the measures above reveals some of our approach's limitations. One disadvantage of our technique is that it necessitates additional computing resources. In the future, we will consider implementing model pruning and employing lightweight models to reduce the computing resources used by the model and further increase performance while keeping lightweight models.

**Table 3:** Comparison of the FLOPs (G) of our method with other methods.

Method	FLOPs
YOLOv5l	107.8
DE-YOLO	87.6
DLN-YOLO	678.5
IAT-YOLO	126.1
Ours	219.5

**Figure 8:**
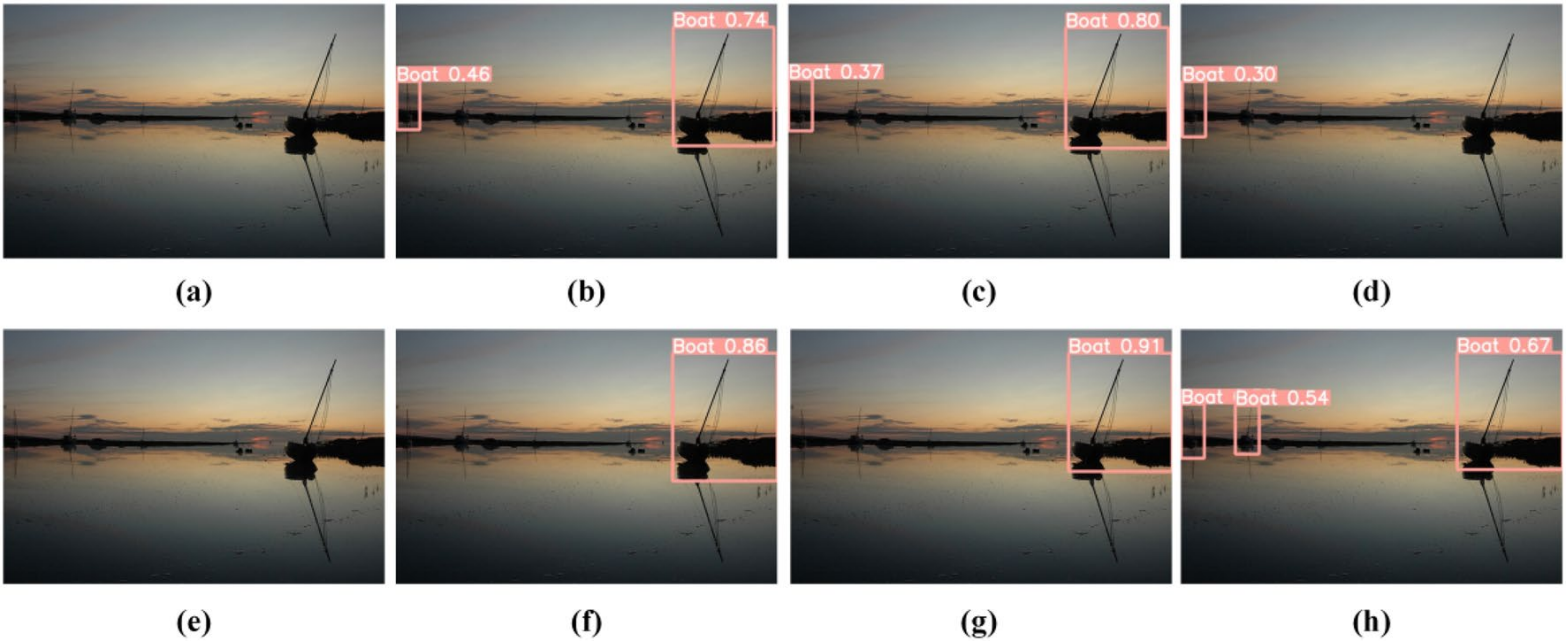
Comparison of alternative image enhancement methods. (**a**) Input, (**b**) YOLOv5l, (**c**) URetinexNet + YOLOv5l, (**d**) SGZ + YOLOv5l, (**e**) DRBN + YOLOv5l, (**f**) URetinexNet + NLE-YO-LO, (**g**) DRBN + NLE-YOLO, (**h**) Ours.

**Figure 9:**
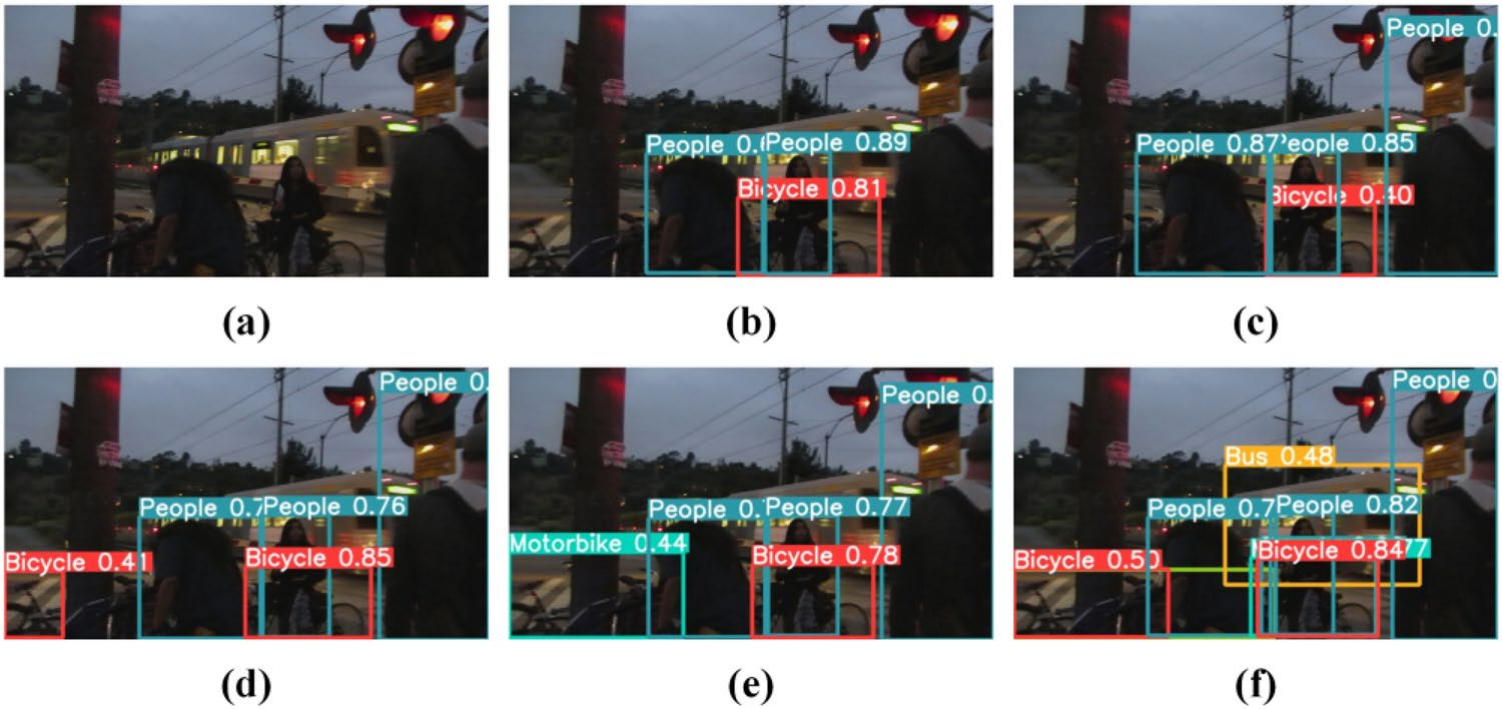
Comparison with other state-of-the-art methods. (**a**) Input, (**b**) YOLOv5, (**c**) DE-YOLO, (**d**) DLN-YOLO, (**e**) IAT-YOLO, (**f**) Ours.

Qualitative comparison. To demonstrate our method's superior performance, we have included some representative photographs to compare with other cutting-edge methods. Figures 8 and 9 highlight this in particular.

As illustrated in Fig. 8, our technique outperforms existing augmented models in terms of performance. The figure clearly shows that there are missed detections in other ways, particularly in the method combined with DRBN, where no target is discovered. In contrast, our technique can detect all items in their entirety. This could benefit from our model's ability to extract rich semantic characteristics while removing the influence of noise.

We quantitatively compared our method to other detection methods to further validate its efficiency. Figure 9 depicts the outcome. The figure clearly shows that our technique has good detection performance and can accurately recognize the item bus in the figure, whereas other methods fail to detect the bus. This totally proves the effectiveness of our method.

### Ablation experiment

We conduct ablation experiments for a complete comparison to verify the impact of different modules on model performance. × indicates that no module is used, while √ indicates that a module is utilized. In the experiments in this section, we start with the YOLOv5l model as the baseline model and gradually add additional modules on top of it to demonstrate the importance of different modules and their impact on model performance. Table 4 displays the outcomes of the ablation trials.

**Table 4:**
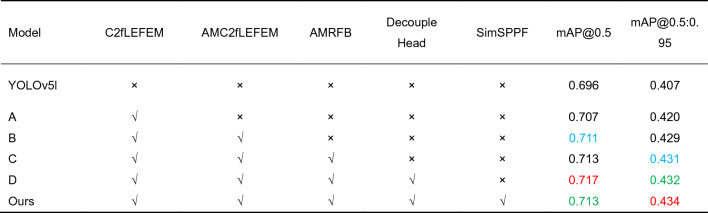
Ablation experiment results (Higher metric values reflect higher performance. Red, green, and blue represent the top-three indicator values, respectively).

Table 4 demonstrates that we receive the best experimental results when all modules are employed, indicating that each module we use is essential for our strategy. Following the addition of the C2fLEFEM module, all model indicators improved, with the value of mAP@0.5 increasing by 1.1% and the value of mAP@0.5:0.95 increasing by 1.3%. This effectively demonstrates that this module can increase the model's feature extraction capability. The AMC2fLEFEM module enhanced the values of mAP@0.5 and mAP@0.5:0.95 by 0.4% and 0.9%, respectively. This demonstrates that the module can direct the network to retrieve more significant feature information. Following the addition of AMRFB, the values of mAP@0.5 and mAP@0.5:0.95 increased by 0.2%. The index improvement demonstrates that the AMRFB module can efficiently extract the features of low-light images while extending the receptive field. With the introduction of the Decouple Head module, the value of mAP@0.5 grew from 0.713 to 0.717, while the value of mAP@0.5:0.95 increased from 0.431 to 0.432. This module's requirement has been demonstrated. The index value was effectively enhanced once the SimSPPF module was introduced to replace the original SPPF module. The following trials convincingly demonstrate the necessity and usefulness of each module, as well as the superiority of our method.

**Figure 10:**
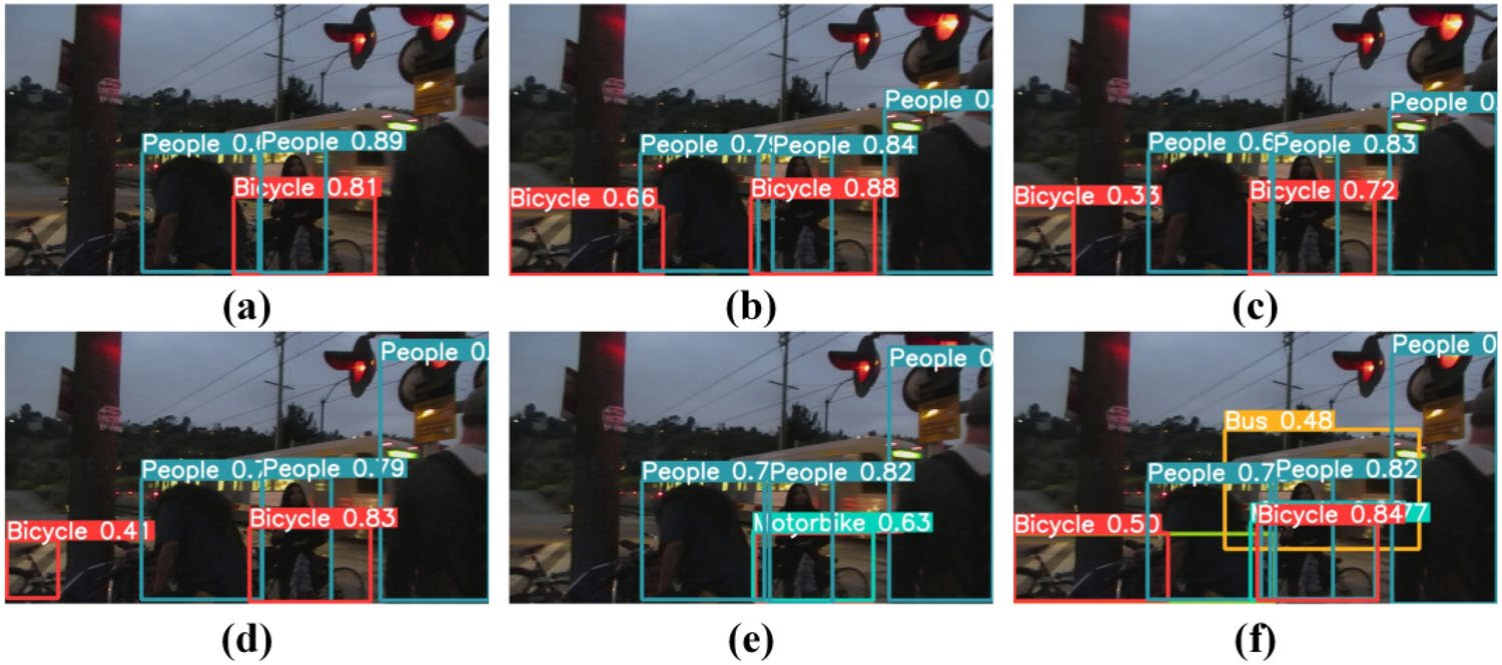
Comparison of ablation experiments. (**a**) YOLOv5l, (**b**) A, (**c**) B, (**d**) C, (**e**) D, (**f**) Ours.

Figure 10 shows some typical ablation experiments to demonstrate the superiority of our technology and the effectiveness of each module. As illustrated in Fig. 10, our approach is the most comprehensive. It demonstrates that our method outperforms other methods in terms of detection performance.

## Conclusion

To address the existing issues with low-light object detection and the noise created during the enhancement process, we offer NLE-YOLO, a revolutionary low-light object detection model. For improved results, we use the augmented image as input to extract more information. We propose a novel feature extraction module, C2fLEFEM, and replace the C3 module in the original YOLOv5 network with this module to reduce high-frequency noise and increase critical information. Furthermore, we suggest a new Attentional Receptive Field Block (AMRFB). This module may broaden the receptive field and assign different attention domains to distinct receptive fields, thus boosting the network's feature extraction ability. We replaced the SPPF module with the SimSPPF module for faster inference and better feature representation. Furthermore, we modified the C2fLEFEM feature extraction module into the AMC2fLEFEM module, which extracts features of different scales by incorporating a simple attention module (SimAM) to increase the network's feature learning capacity. Finally, to make the network more appropriate for low-light conditions, we replaced the initial detection head with a decoupled head. Experiments are carried out on the Exdark dataset, and quantitative and qualitative findings reveal that our method beats state-of-the-art methods and has higher detection accuracy.

## Data Availability

The datasets analysed during the current study are a-vailable at https://github.com/cs-chan/Exclusively-Dark-Ima-ge-Dataset.
